# 4236 Sleep Disorders and Diabetic Complications

**DOI:** 10.1017/cts.2020.147

**Published:** 2020-07-29

**Authors:** Magda Shaheen, Meleesa Nocera, Senait Teklehaimanot

**Affiliations:** 1 David Geffen School of Medicine at UCLA; 2 Charles R Drew University

## Abstract

OBJECTIVES/GOALS: Diabetes is a prevalent chronic illness that imposes health-related burdens including nephropathy, retinopathy and sleep disorders. The goal of this study was to examine the relation between both sleep disorders and sleep duration and diabetic chronic kidney disease (CKD) and retinopathy. METHODS/STUDY POPULATION: We analyzed data from the National Health and Nutrition Examination Survey 2005-2016 related to diabetic nephropathy and retinopathy, sleep disorders and duration, demographics, and risk factors among diabetics. The subjects were adults with diabetes type 2. Multiple logistic regression analysis was performed to look at the relationship between diabetic complications (CKD and retinopathy) and sleep disorders and sleep duration adjusting for demographics and risk factors. RESULTS/ANTICIPATED RESULTS: Of the 4087 diabetics, 45% had CKD, 19% had retinopathy, and 15% had sleep disorders. CKD and retinopathy were not associated with sleep disorders (p>0.05) but CKD was associated with sleep duration (Adjusted odds ration = 1.014, 95% confidence interval = 1.001-1.027, p<0.05). Cardiovascular disease was a predictor of both CKD and nephropathy (P<0.05). Other predictors of CKD and nephropathy were age >60 years, Non-Hispanic Black, hypertension, low education level, and living under 200% of the Federal Poverty Level (P<0.05). DISCUSSION/SIGNIFICANCE OF IMPACT : Among diabetics, CKD and retinopathy were not associated with sleep disorders, and only CKD was associated with sleep duration. These findings may impact the management of diabetes in the future, since it has effects on a range of other health conditions.

